# Supplementary material for: Co-Creation of a School-Based Motor Competence and Mental Health Intervention: Move Well, Feel Good
Source: Children (Basel). 2023 Aug 17;10(8):1403. doi: 10.3390/children10081403 (PMC10453743; doi:10.3390/children10081403)
Supplement: Supplementary file 1 [file children-10-01403-s001.zip › children-2527555-supplementary.pdf]

Supplement File S1. School recruitment video.

https://bit.ly/3uoYsnC

Figure S1. Activity prompt cards example

Wobble Spot

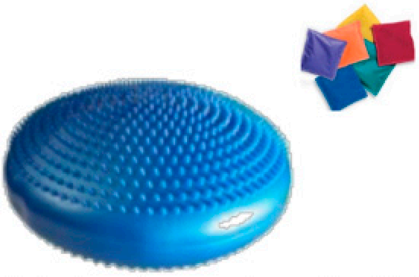

1. Pick the bean bag up, get yourself set, that means get your balance on one leg (try your strongest leg or the one you kick with)
2. Focus on something in front of you
3. Stand up tall when you balance
4. Pass the bean bag around your tummy in a circular motion five times

- Don't worry, try your best
- *Count out loud in encouragement*
- Keep going
- Never mind, well done
- Great job
- Excellent

Overarm Throw

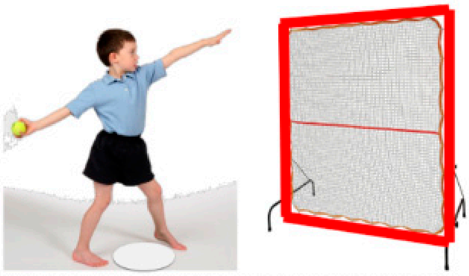

1. Look at the target and focus on hitting the net with the ball
2. Swing your arm down and back as you prepare to throw and point to the target (with your non-throwing arm)
3. Stand side-on, take a step forward, throw, and follow-through down and across your body

- Don't worry, try your best
- You can do it
- Excellent/Good throw!
- Hit!! Well done!!
- Nearly, so close, never mind, well done

Table S1. Research evidence presented to adult stakeholders in workshop 3.

**Intervention focus**	**Delivery context**	**Delivery personnel**	**Pedagogical considerations**	**Intervention dose**	**Other considerations**
Specific aspects of FMS	PE lessons	PE/PA specialists	Mastery learning climate (SAAFE Framework, TARGET structure)	At least 1 taught session per week embedded into curriculum	Training for deliverers in some but not all effective interventions
Socio-emotional de velopment	Active play (break times)	Generalist teachers	Assessment for Learning	60-90 minutes per session	Limited evidence in children aged 9-10 (nearest is age 8: A+FMS programme)
	Classroom for socio-emotional develop ment focus	Coaches	Use of additional resources for independent practice (e.g., pupil handbook, digital resources)	Additional self-directed opportunities (e.g., breaks, home)	
	Home	Self/peers			

Figure S2. Co-production interview guide

Introduction

- Thank the participant for taking part in the interview.
- Describe the purpose of the interview in relation to the Move Well Feel Good project
- Key components of the interview
  - What your thoughts were on the co-production process
  - Positives and negatives of the experience
  - Feedback
- Confidentiality procedures
  - I'd like to tell you that I will be recording the conversation to help me remember what you said.
  - You can ask for the recording to be stopped at any time.
  - After the interview, the recording will be transcribed/written up word for word.
  - After the interview has been transcribed, the audio recordings will be destroyed.
  - In the transcripts, all identifying information will be removed, so nothing you say can be connected to you in any way.
  - Do you have any questions for me?
  - Are you happy to proceed with the interview?

Questions

1. Were the workshop tasks and activities easy to understand and complete?
   a) Workshop 1 – Online knowledge and understanding
   b) Workshop 2 – Face-to-face
   c) Workshop 3 – Edge Hill University draft and development of intervention ideas
   d) Workshop 4 – feedback of ideas – consensus vote
      - What are your thoughts on the final idea that is being rolled out?
      - The overall value/benefit of the process?

2. What did you particularly enjoy about being involved in the co-production process?
3. Is there anything that you think could have been done better? If so, what were these?
4. How did you feel that the communication between the research team and yourselves was throughout the process?
5. How did you find the group dynamics throughout the face to face workshops? Did you feel like you were able to contribute in the way you wanted?
6. Do you have any more comments or feedback on things that I haven't mentioned?

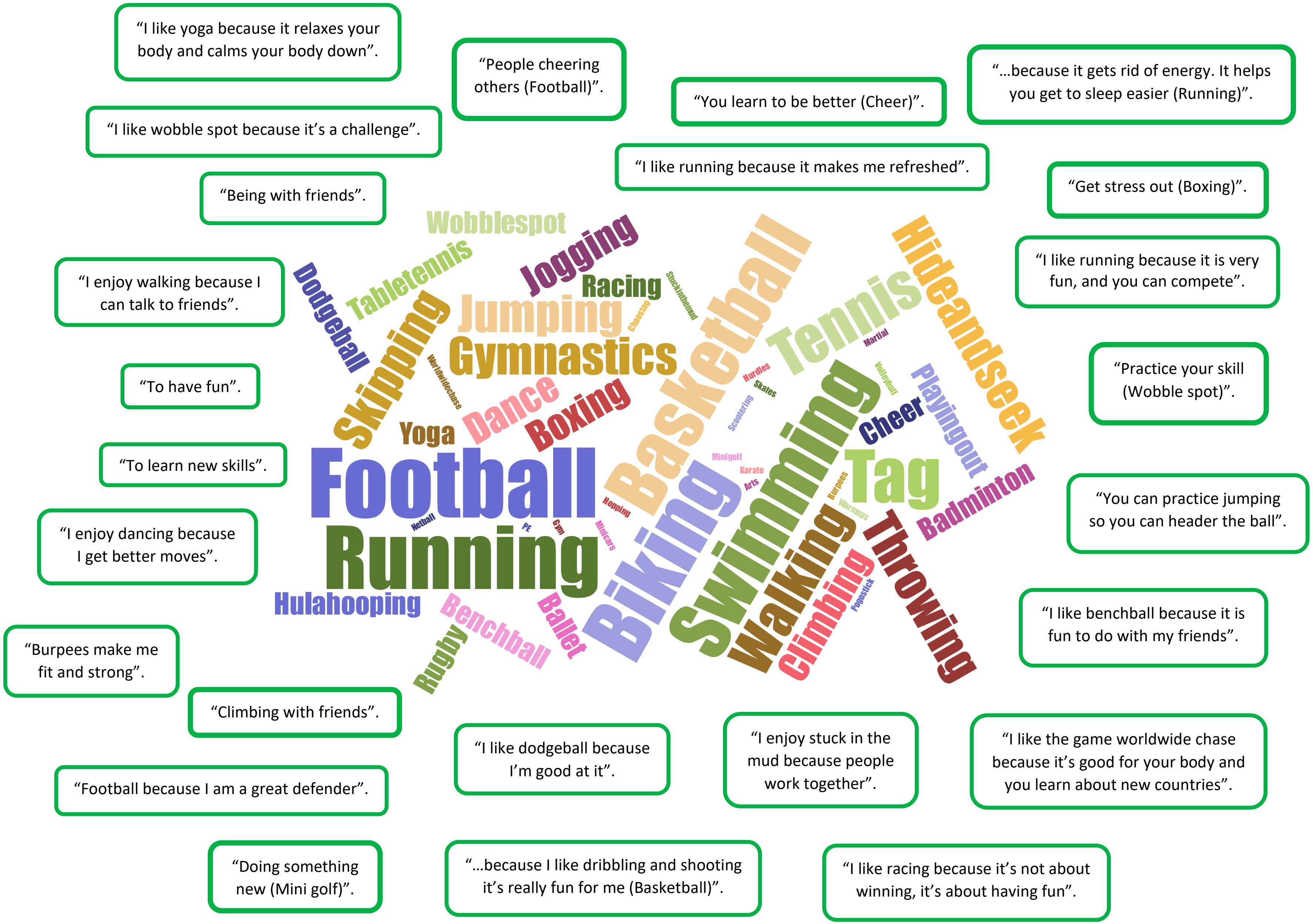

Figure S3. Activities that children reported to enjoy and the reasons why.

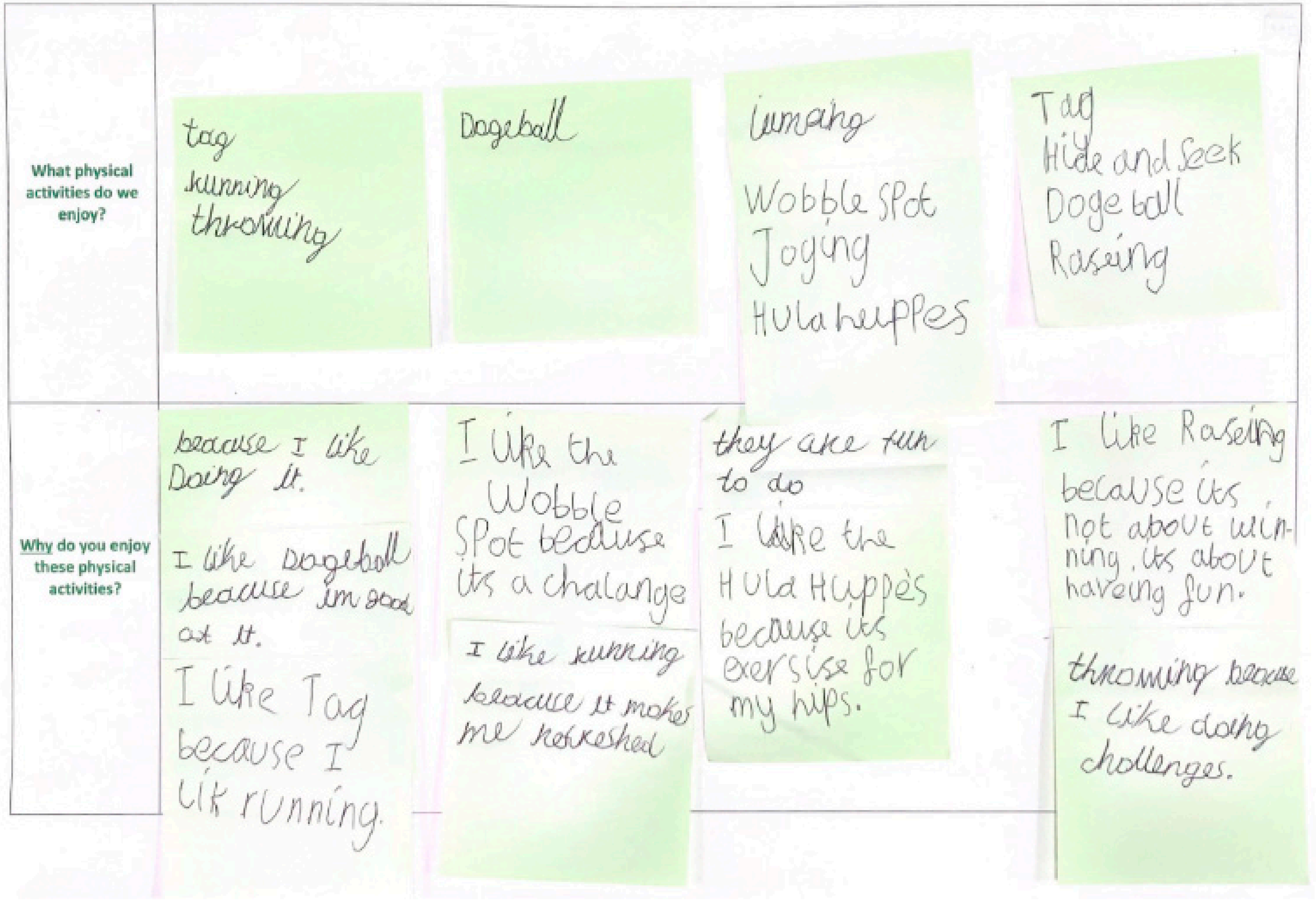

Figure S4. Example of children's work from workshop 2.

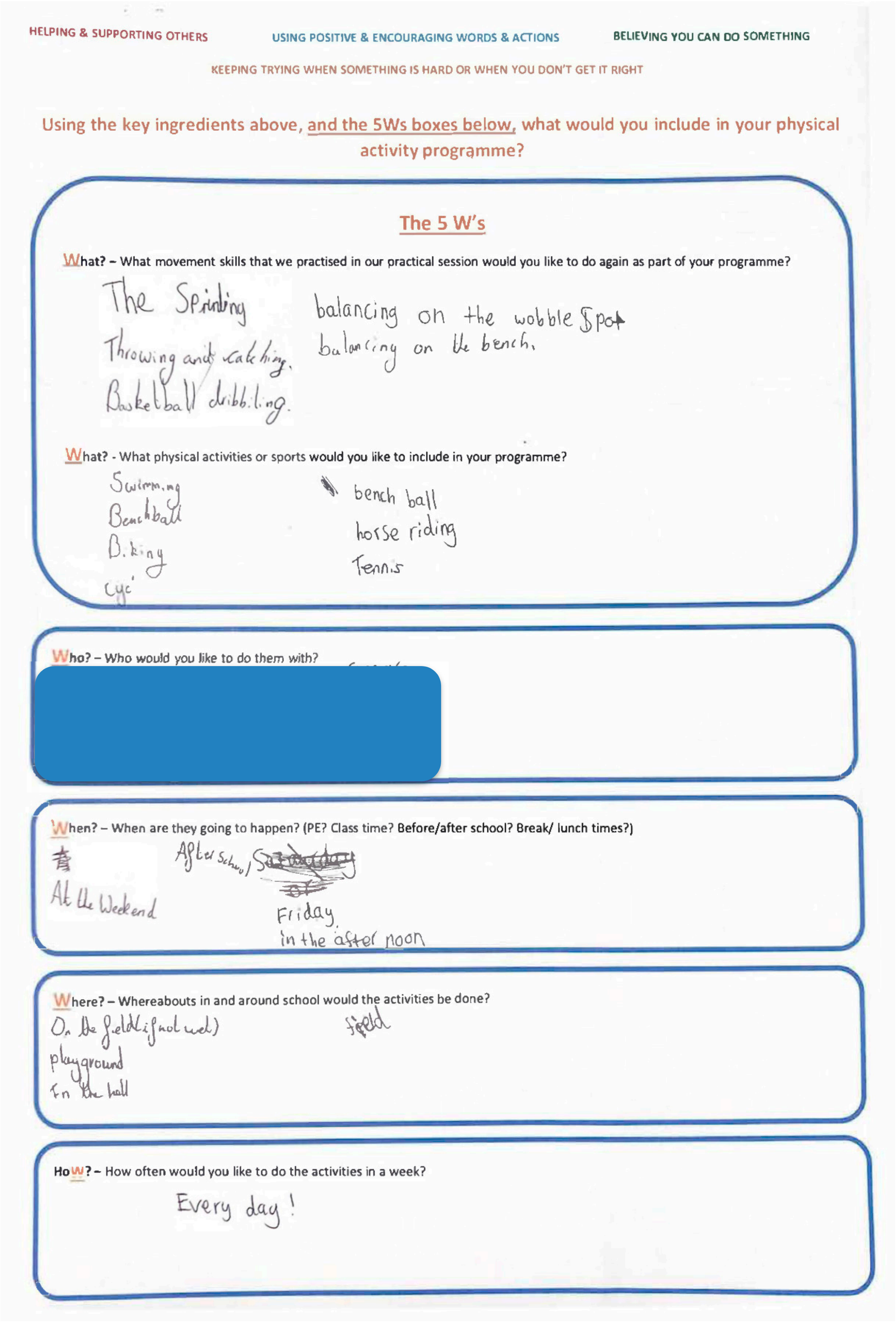
HELPING & SUPPORTING OTHERS

USING POSITIVE & ENCOURAGING WORDS & ACTIONS

BELIEVING YOU CAN DO SOMETHING

KEEPING TRYING WHEN SOMETHING IS HARD OR WHEN YOU DON'T GET IT RIGHT

Using the key ingredients above, and the 5Ws boxes below, what would you include in your physical activity programme?

The 5 W's

What? – What movement skills that we practised in our practical session would you like to do again as part of your programme?

The Spinning
Throwing and catching.
Basketball dribbling.
balancing on the wobble spot
balancing on the bench.

What? - What physical activities or sports would you like to include in your programme?

Swimming
Benchball
Biking
cyc
bench ball
horse riding
Tennis

Who? – Who would you like to do them with?

[redacted]

When? – When are they going to happen? (PE? Class time? Before/after school? Break/ lunch times?)

At the Weekend
After School ~~Saturday~~ ~~or~~
Friday
in the after noon

Where? – Whereabouts in and around school would the activities be done?

On the field (if not wet)
playground
In the hall
field

HoW? – How often would you like to do the activities in a week?

Every day!

Figure S5. Example of children's work from workshop 2.
